# Associations Between Historically Redlined Districts and Racial Disparities in Current Obstetric Outcomes

**DOI:** 10.1001/jamanetworkopen.2021.26707

**Published:** 2021-09-30

**Authors:** Stefanie J. Hollenbach, Loralei L. Thornburg, J. Christopher Glantz, Elaine Hill

**Affiliations:** 1 Division of Maternal Fetal Medicine, Department of Obstetrics and Gynecology, University of Rochester Medical Center, Rochester, New York; 2 Department of Biomedical Engineering, University of Rochester, Rochester, New York; 3 Department of Public Health Sciences, University of Rochester Medical Center, Rochester, New York

## Abstract

**Importance:**

Despite much higher health care expenditure than comparable countries, striking racial and ethnic disparities exist in obstetric outcomes in the United States. A multifaceted exploration of the factors influencing these disparities, including the legacy of structural racism, is important to improve health outcomes for all.

**Objective:**

To characterize the association of the historic racially discriminatory home loan practice of redlining with disparities in modern obstetric outcomes.

**Design, Setting, and Participants:**

In this retrospective cohort study of a 9-county birth certificate database in the Finger Lakes region of New York state from 2005 to 2018, modern obstetric outcomes were matched with regions classified by the federal government for mortgage loan servicing based on racially discriminatory criteria from the 1940 Home Owners’ Loan Corporation map (HOLC; also known as *the redline map*). Patients with a live birth recorded in the data system with a recorded home zip code within the historic HOLC categories were included. Data were analyzed from July to December 2019.

**Exposure:**

Regions previously categorized by historic, racially discriminatory criteria.

**Main Outcomes and Measures:**

Each HOLC area was analyzed for the primary outcome of preterm birth and secondary outcomes of obstetric and medical complications, with logistic regression to address regional and patient-level covariates.

**Results:**

From 2005 until 2018, there were 64 804 live births within the 15 zip codes overlaying historic HOLC regions. Prevalence of preterm birth increased with decreasing HOLC categories, from the lowest overall preterm birth rate of 217 of 2873 births (7.55%) in the zip code historically defined as “Best” or “Still Desirable” and the highest overall preterm birth rate of 427 of 3449 births (12.38%) in the zip code historically defined as “Hazardous.” These associations with preterm birth remained significant in logistic regression controlling for poverty levels and educational attainment (adjusted odds ratio, 1.46; 95% CI, 1.08-1.97) and parental race (adjusted odds ratio, 1.38; 95% CI, 1.25-1.53).

**Conclusions and Relevance:**

In this cohort study, the linkage of historic and modern community data sets with an obstetric data set offered the opportunity to characterize modern obstetric disparities associated with a system of historic inequity. The persistence of these findings after correcting for contemporary community socioeconomic characteristics suggest potential influences of a system of profound structural inequity that ripple forward in time, with impacts that extend beyond measurable socioeconomic inequity.

## Key Points

**Question:**

Is the legacy of structural racism in the housing market associated with disparate outcomes in a contemporary obstetric cohort?

**Findings:**

In this cohort study including 64 804 live births in 17 zip codes, historically redlined zip codes were associated with increased risk of preterm birth and periviable birth.

**Meaning:**

These findings suggest that historic discriminatory policies remain associated with poor obstetrical outcomes, suggesting intergenerational discrimination and disinvestment continue to have a role in modern health disparities.

## Teaser

This cohort study examines the associations of the historic racially discriminatory home loan policy of redlining with modern obstetric outcomes in a US city.

## Introduction

The US has higher infant and maternal mortality rates than nearly any other Organization for Economic Cooperation and Development nation despite also having the largest health expenditures.^1,2^ Racial and ethnic disparities in outcomes are well documented, with Black women carrying a disproportionate burden of increased morbidity and mortality due to a range of obstetric outcomes.^3,4,5,6^ These disparities are persistent across regions in the US; when statewide pregnancy-related mortality rate is used to group states into low, medium, and high pregnancy-related mortality groups, the risk for Black women is persistently around 3-fold higher than for White women in each risk strata. Additionally, risk of death in pregnancy is higher for Black women with at least a college degree than for White women who have not completed a high school diploma.^7^

These disparities remain pronounced across the spectrum of obstetric experiences. Preterm birth demonstrates particularly prominent racial and ethnic disparities in the US, with non-Hispanic Black women experiencing a preterm birth rate at least 50% higher than non-Hispanic White women.^8^

Increasing focus of the medical community on health care disparities has revealed a wide array of contributions to the inequity of health outcomes, ranging from the physical environment to socioeconomic factors, individual health behaviors, stress, and genetic factors.^8,9,10,11^ The cumulative effect of structural racism is one driver of the disproportionate negative outcomes faced by Black women.^3^ This is the factor that likely gives rise to the so-called *immigrant paradox*, in which immigrant women have better health outcomes on arrival to the US than after multiple years of living within the US.^12^ The legacy of structural racism is extensive in the US^13^; one historic vehicle of discrimination and disinvestment was the practice of redlining: a formal practice of the federal government’s Home Owners’ Loan Corporation (HOLC) beginning in the 1930s to delineate areas where mortgages could be insured based on overtly racially discriminatory criteria.^14^

From the 1930s into the 1940s, the federal government created thousands of area descriptions for cities across the US. First created by the HOLC, redline policies were then adopted by the Federal Housing Administration and the Department of Veterans Affairs, all of which ostensibly used these classifications to guide where it was financially “safe” to issue mortgages, although “safety” frequently represented a categorization for the racial composition of neighborhoods, with predominantly White neighborhoods categorized in lowest risk categories (ie, grades A and B).^15^ This structure of disinvestment, which formally stretched forward into the 1960s, has far reaching impacts. Owing to a range of sequelae of community disinvestment, as well as the critical role of home ownership in intergenerational wealth-building, the legacy of historic redline racial discrimination correlates with modern social and health inequities.^3,14,15,16,17^ We conducted this study to evaluate our hypothesis that the legacy of racial discrimination measured by historic redlining is associated with modern obstetric outcome disparities.

## Methods

This cohort study was approved by the University of Rochester Research Subjects Review Board, including a waiver of informed consent consistent with institutional review board approval for use of deidentified data. This study is reported following the Strengthening the Reporting of Observational Studies in Epidemiology (STROBE) reporting guideline.

### Study Design

We performed a retrospective cohort study of patients with live births from 2005 until 2018 in the Finger Lakes Region perinatal and obstetric data system—a New York State Department of Health electronic birth certificate database. This includes the city of Rochester, New York, in Monroe county, plus 8 counties south and east of Monroe county, known as the Finger Lakes region.

### Outcome and Covariate Data

Deidentified data, with 5-digit zip codes retained, from the perinatal and obstetric data system were tabulated for obstetric outcomes and patient demographic characteristics, including race. In the obstetric data system, patient race is collected from self-identification on the data collection form filled out by the patient at the time of the delivery hospitalization.

Zip codes with at least 100 deliveries during the study period were stratified into cohorts, and frequencies of patient-level obstetric outcomes were calculated for each. Zip codes with fewer than 100 deliveries over the study period were excluded to avoid introducing confounding from small groups.

Preterm birth is defined as any gestation length less than 37 weeks. We also included extreme preterm birth (ie, births <28 weeks) and periviable birth (births at 22-25 weeks), as these births impart the greatest risks for neonatal morbidity and mortality and are thus of particular focus. Secondary outcomes of interest included maternal diagnoses of pregnancy-related hypertension, severe depression, and substance use and additional pregnancy-related outcomes, including neonatal intensive care unit admission, 5-minute APGAR score less than 7, and exclusive breastfeeding in the immediate postpartum period.

The data set then was merged with publicly available community data from the US Census Bureau. Zip code cohorts were linked to income, poverty, and educational attainment data, as described by the US Census Bureau based on the 2017 American Community Survey.^18^ Median gross income of community members, percentage of community members with a high school diploma, and percentage of community members living below the US poverty level were tabulated for each zip code.

### Defining Exposure

**Figure 1.**
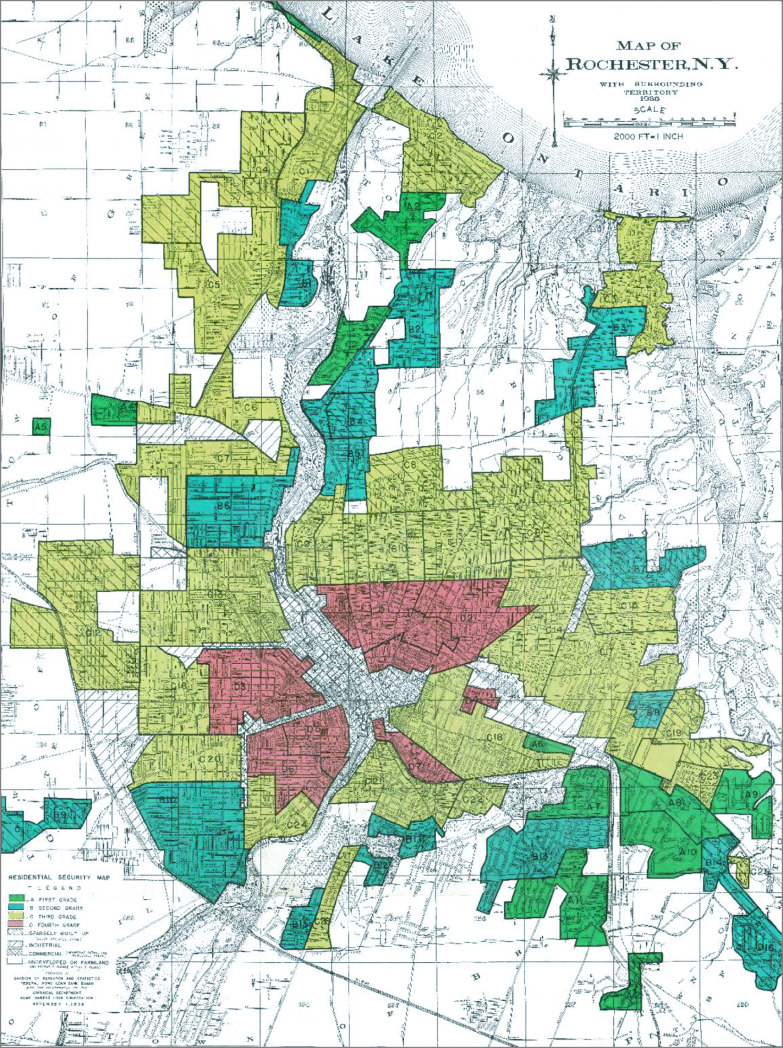
Historic Redlining Map of the City of Rochester Source: Mapping Inequity initiative, as licensed under a Creative Commons Attribution.^14^ Red areas indicate most “Hazardous,” while green areas are “Best.”

We categorized each zip code in our birth registry based on the 1940 HOLC map of the city of Rochester^14^ to classify them according to the classification by the federal government as A, “Best”; B “Still Desirable”; C, “Definitely Declining”; and D, “Hazardous” for mortgage loan servicing based on racially discriminatory criteria (Figure 1; eFigure 1 in the Supplement). Assignment to category was based on direct visual overlay with the modern zip code regions to correlate these zip codes with either a single historic designation or potentially 2 or more historic designations because modern zip codes geography differs from these historic regions. Zip code cohorts were separately included in the statistical analysis if they contained 3 or more historic designations to avoid confounding from modern zip codes that contained many types of historic designations.

### Statistical Analysis

Our unit of analysis was live births clustered within zip codes that were coded by HOLC grades. We used logistic regression with a random effect for each zip code and clustered SEs at the zip code level to account for correlation within zip code. We then adjusted separately for community economic factors (poverty levels, categorized as <10%, 10 to <20%, or ≥20% of community members living at or below the federal poverty level, and educational attainment, categorized as <75%, 75% to <85%, or ≥85% of community members graduated from high school) and maternal and paternal race.

Stata statistical software version 16.0 (StataCorp) was used to collapse the obstetric data set into zip code cohorts to describe geographic distribution of outcomes as well as to perform the statistical analyses. Graphic representations of the distributions of obstetric, and community socioeconomic data were generated in ArcGISPro version 2.5.0 (Esri). *P* values were 2-sided, and statistical significance was set at *P* = .05. Data were analyzed from July to December 2019.

## Results

From 2005 until 2018, the birth data system contained detailed information for 210 984 live births. A total of 199 088 live births were eligible for inclusion as being associated with a zip code containing more than 100 deliveries in the study period with resulting 120 zip code cohorts.

Of these births, there were 64 804 within the 15 zip codes that were redlined and had HOLC grades in Rochester, New York. One contemporary zip code overlapped with the historic D “Hazardous” designation, and 1 contemporary zip code overlapped with historic regions characterized as A “Best” or B “Still Desirable.” Ten zip codes were distributed in 1 or 2 categories between these extremes, and 3 contained 3 or more of the historic designations and thus were tabulated but not included in the statistical analysis.

**Figure 2.**
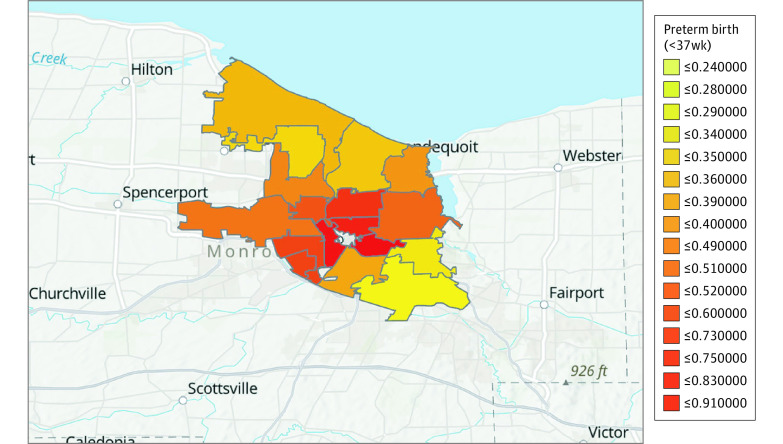
Incidence of Any Preterm Birth in the Modern Zip Code Regions Overlaid on the Historic Redlining Map Darker red represents the highest preterm birth rates and yellow represents the lowest preterm birth rates.

Prevalence of preterm birth increased with worse HOLC grades, with the lowest overall preterm birth rate of 217 of 2873 births (7.55%) in the zip code historically defined as “Best” or “Still Desirable” and the highest overall preterm birth rate of 427 of 3449 births (12.38%) in the zip code historically defined as “Hazardous.” Overall preterm birth, extreme preterm birth, and periviable birth by HOLC designation are presented in Table 1. The rate of periviable birth was 3-fold higher in the “Hazardous” neighborhood than the “Best” or “Still Desirable” neighborhood (26 births [0.75%] vs 7 births [0.24%]) (eFigure 3 and eFigure 4 in the Supplement). Figure 2 depicts concentrations of preterm birth among modern zip code regions and eFigure 2 in the Supplement depicts concentrations of income, poverty, and educational attainment across modern zip code regions. Adjusting for patient-level birth data and with “Best” HOLC designation as the reference category, there was a graded increase in preterm birth with worsening HOLC designation, with adjusted odds ratios of 1.19 (95% CI, 1.08-1.31) for “Definitely Declining” (*P* = .001) to 1.38 (95% CI, 1.25-1.53) for “Hazardous” (*P* < 001) (Table 2).

**Table 1.** Preterm Birth Incidence Organized by Historic HOLC Designation

Historic HOLC designation	No.		Preterm birth, No. (%)		
	Zip codes	Births	Any	<28 wk	Periviable birth
“Best” or ”still desirable”	1	2873	217 (7.55)	14 (0.49)	7 (0.24)
“Still desirable” or “definitely declining”	5	27 947	3113 (11.14)	362 (1.30)	163 (0.58)
“Definitely declining”	3	14 542	1407 (9.68)	132 (0.91)	58 (0.40)
“Hazardous” or “definitely declining”	2	6180	736 (11.91)	100 (1.62)	42 (0.68)
“Hazardous”	1	3449	427 (12.38)	47 (1.36)	26 (0.75)
Currently contain ≥3 designation regions	3	9813	813 (8.28)	69 (0.70)	33 (0.34)
Total cohort, %	15	64 804	10.36	1.12	0.51
Regional population, %	120	199 088	9.48	0.77	0.36

Abbreviation: HOLC, Home Owners’ Loan Corporation.

**Table 2.** Progressive Multivariable Regression Analysis of All Preterm Births in Historically Redlined Regions Compared With the “Best” or “Still Desirable” Historic Regions

Historic HOLC designation	Unadjusted regression		Adjusted regression			
	OR (95% CI)	*P* value	Community economics		Maternal and paternal race	
			OR (95% CI)	*P* value	OR (95% CI)	*P* value
“Still desirable” or “Definitely declining”	1.48 (1.37-1.61)	<.001	1.41 (1.10-1.81)	.007	1.28 (1.18-1.39)	<.001
“Definitely declining”	1.32 (1.18-1.48)	<.001	1.38 (1.08-1.75)	.01	1.19 (1.08-1.31)	.001
“Hazardous” or “Definitely declining”	1.54 (1.10-2.15)	.03	1.45 (1.01-1.97)	.04	1.33 (1.12-1.56)	.001
“Hazardous”	1.73 (1.62-1.85)	<.001	1.46 (1.08-1.97)	.01	1.38 (1.25-1.53)	<.001
Currently contain ≥3 designation regions	1.09 (1.00-1.19)	.03	1.10 (0.92-1.31)	.30	1.07 (0.93-1.22)	.34

Abbreviations: HOLC, Home Owners’ Loan Corporation; OR, odds ratio.

Geographic disparities in modern preterm birth rates across the entire region remained statistically significant in multivariable regression controlling for community-level poverty levels and educational attainment and patient-level maternal race and paternal race in rates of all preterm births (Table 2 and Table 3). Thus, even when controlling separately for community and patient-level characteristics, patients living in the “Hazardous” region had higher odds of any preterm birth (community-level adjusted OR, 1.46; 95% CI, 1.08-1.97; patient-level adjusted OR, 1.38; 95% CI, 1.25-1.53). This association persisted across the most heavily redlined groups. For periviable birth, women living in the “Hazardous” region had highest odds in unadjusted models (OR, 3.11; 95% CI, 3.11-3.11), with modest attenuation after controlling for community resource characteristics (adjusted OR, 2.16; 95% CI, 1.56-2.98) and patient-level race (adjusted OR, 1.74; 95% CI, 5 1.37-2.21). In contrast, the association of periviable birth with higher HOLC grade were no longer statistically significant after multivariable regression controlling for patient-level characteristics, although they remained significant when controlling for community-level characteristics.

**Table 3.** Progressive Multivariable Regression Analysis of Periviable Birth in Historically Redlined Regions Compared With the “Best” or “Still Desirable” Historic Regions

Historic HOLC designation	Unadjusted regression		Adjusted regression			
	OR (95% CI)	*P* value	Community economics		Maternal and paternal race	
			OR (95% CI)	*P* value	OR (95% CI)	*P* value
“Still desirable”or- “definitely declining”	2.40 (1.99-2.89)	<.001	1.90 (1.58-2.28)	<.001	1.46 (1.12-1.89)	.005
“Definitely declining”	1.64 (1.26-2.14)	<.001	1.41 (1.04-1.91)	.03	1.20 (0.92-1.56)	.20
“Hazardous” or “definitely declining”	2.80 (2.15-3.65)	<.001	2.06 (1.20-3.53)	.008	1.56 (0.85-2.86)	.16
“Hazardous”	3.11 (3.11-3.11)	<.001	2.16 (1.56-2.98)	<.001	1.74 (1.37-2.21)	<.001
Currently contain ≥3 designation regions	1.38 (1.26-1.51)	<.001	1.31 (1.17-1.47)	<.001	1.22 (1.06-1.39)	.005

Abbreviations: HOLC, Home Owners’ Loan Corporation; OR, odds ratio.

When comparing secondary outcomes between the extremes of HOLC grades, there was a significant difference in all measured secondary complications and outcomes (eTable in the Supplement). This correlated with a particularly prominent increased odds of severe maternal depression (OR, 4.40; 95% CI, 3.15-6.17) and a diagnosed substance use disorder (OR, 16.37; 95% CI, 5.97-22.40) as well as a decreased odds of exclusive breastfeeding at the time of hospital discharge (OR, 0.12; 95% CI, 0.11-0.13).

## Discussion

This cohort study found associations between modern obstetric outcome disparities and historic policies driving intergenerational inequity based on race. Our findings provide further evidence of the influence of a legacy of structural racism on the disproportionate burden of adverse pregnancy outcomes for Black women in the US.

In our study population of a single midsized US city, historic redlining was associated with increased risk of preterm birth in the modern day. This association between historic redlining and contemporary preterm birth rates is striking across the gestational age range. The historic redlining was associated with contemporary increased odds of any preterm birth that did not resolve when factoring in community or patient-level characteristics, particularly in the regions historically designated with the greatest “Hazard.” The persistence of the association of any preterm birth with increasing “Hazard” designation by the HOLC, even when accounting for contemporary community income levels, poverty levels, and educational attainment, suggests that current community resource distribution alone does not explain these disparities in outcome. Potentially, the overarching influence of a system of profound structural inequity ripples forward in time with impacts that extend beyond measurable socioeconomic inequity.

In contrast, when analyzing the most extreme forms of preterm birth, the unadjusted risk of periviable birth with decreasing HOLC grade ceased to be statistically significant when we controlled for patient-level racial characteristics but not when we controlled for community-level economic characteristics. This finding may generate new hypotheses surrounding the relative association of social and environmental factors across the spectrum of preterm birth, including the roles that income, insurance access, and life stressors may play in the pathogenesis of preterm birth. Moreover, future studies may have the opportunity to explore the potentially differential associations of these factors with preterm births that occur spontaneously vs those that are iatrogenically created owing to medically indicated deliveries.

When we compared secondary outcomes between the extremes of historic HOLC designation, giving birth when living in the regions historically designated with the greatest “Hazard” was associated with contemporary increased odds of experiencing a range of pregnancy-related comorbidities, as well as increased odds of adverse obstetric outcomes. Odds of severe maternal depression and a diagnosis of substance use disorder were markedly increased among women delivering from the historically “Hazardous” area. By the time of their delivery, these women then also experienced increased odds of maternal complications, such as pregnancy-related hypertension, and neonatal complications, such as low APGAR scores and higher rates of neonatal intensive care unit admission. These associations underscore the echoes of the past—imparting higher morbidity for members of communities impacted by a legacy of disinvestment.

### Limitations

This study has some limitations. Our study characterized the modern impacts associated with historic, racially discriminatory policy in a single, midsized US city. Given the size of the region, the extremes of HOLC designation included only 1 zip code each, which limits our power and may introduce additional unidentifiable confounders in our multivariable regression. Findings from this single region may also not be applicable to other regions in the US.

These data have the possible limitations of all large data sets, including the potential for inaccurate data and residual confounding, although the extensive training and codification of birth certificate data in our system lessens these risks. Moreover, owing to the fact that this is a registry of live births, the impact of the assessed factors by definition did not include stillbirths.

While limited in scope, our study design provides the opportunity to perform an in-depth analysis that integrated community-level data into a data set of patient-level obstetric outcomes, superimposed on historic HOLC designations. This reinforces the findings of a 2020 study delineating associations between historical redlining and birth outcomes in a modern cohort of deliveries in California^19^ and also adds the opportunity to integrate zip code–level economic and educational attainment data into the regression models, independent of patient-level socioeconomic data.

## Conclusions

This cohort study integrated historic and modern community data sets into obstetric data sets, offering the opportunity to analyze the contemporary health legacy of historic inequity. Given the sweeping impacts of disproportionate burden of adverse pregnancy outcomes on Black women and their families associated with historical racially discriminatory housing policy, attempts to dismantle these disparities must be multifaceted and informed by increasingly integrated analysis of previously disparate data sets. The observation that racially discriminatory home lending patterns of the 1940s were associated with contemporary preterm birth rates can inform us that the legacy of government-sanctioned discrimination persists today.

## Notes

Supplement. **eTable.** Secondary Outcome Associations Between the Extremes of Historic HOLC Designation and Pregnancy Complications or Outcomes **eFigure 1.** Modern Zip Code Distributions Overlaid on the Historic HOLC Map of Rochester, New York **eFigure 2.** Distribution of Community Resources **eFigure 3.** Incidence of Any Preterm Birth as by Historic HOLC Designation **eFigure 4.** Incidence of Extreme Preterm Birth and Periviable Birth by Historic HOLC Designation
