# Supplementary material for: Associations Between Historically Redlined Districts and Racial Disparities in Current Obstetric Outcomes
Source: JAMA Netw Open. 2021 Sep 30;4(9):e2126707. doi: 10.1001/jamanetworkopen.2021.26707 (PMC8485176; doi:10.1001/jamanetworkopen.2021.26707)
Supplement: Supplement. — eTable. Secondary Outcome Associations Between the Extremes of Historic HOLC Designation and Pregnancy Complications or Outcomes eFigure 1. Modern Zip Code Distributions Overlaid on the Historic HOLC Map of Rochester, New York eFigure 2. Distribution of Community Resources eFigure 3. Incidence of Any Preterm Birth as by Historic HOLC Designation eFigure 4. Incidence of Extreme Preterm Birth and Periviable Birth by Historic HOLC Designation [file jamanetwopen-e2126707-s001.pdf]

Supplemental Online Content

Hollenbach SJ, Thornburg LL, Glantz JC, Hill E. Associations between historically redlined districts and racial disparities in current obstetric outcomes. *JAMA Netw Open*. 2021;4(9):e2126707. doi:10.1001/jamanetworkopen.2021.26707

eTable. Secondary Outcome Associations Between the Extremes of Historic HOLC Designation and Pregnancy Complications or Outcomes
eFigure 1. Modern Zip Code Distributions Overlaid on the Historic HOLC Map of Rochester, New York
eFigure 2. Distribution of Community Resources
eFigure 3. Incidence of Any Preterm Birth as by Historic HOLC Designation
eFigure 4. Incidence of Extreme Preterm Birth and Periviable Birth by Historic HOLC Designation

This supplemental material has been provided by the authors to give readers additional information about their work.

eTable. Secondary Outcome Associations Between the Extremes of Historic HOLC Designation and Pregnancy Complications or Outcomes

Outcome	OR (95% CI, p)
All categories of preterm birth[a]<br>Preterm birth <28 weeks[a]<br>Periviable birth 22-25 weeks[a]<br>Pregnancy-associated hypertension	1.73 (1.46-2.05, p <0.001)<br>2.82 (1.55-5.13, p = 0.001)<br>3.11 (1.35-7.18, p = 0.008)<br>1.26 (1.03-1.52, p = 0.02)
Severe maternal depression	4.40 (3.15-6.17, p < 0.001)
NICU admission	1.62 (1.38-1.90, p < 0.001)
5 minute APGAR score <7	2.60 (1.69-4.02, p < 0.001)
Substance use	16.4 (6.0-22.4, p < 0.001)
Exclusive breastfeeding	0.12 (0.11-0.13, p < 0.001)

[a]Primary outcomes of preterm birth provided for comparison.

© 2021 Hollenbach SJ et al. *JAMA Network Open.*

eFigure 1. Modern Zip Code Distributions Overlaid on the Historic HOLC Map of Rochester, New York

Supplemental Figure 1: Modern Zip Code Overlay on Historic Red-Lining Map

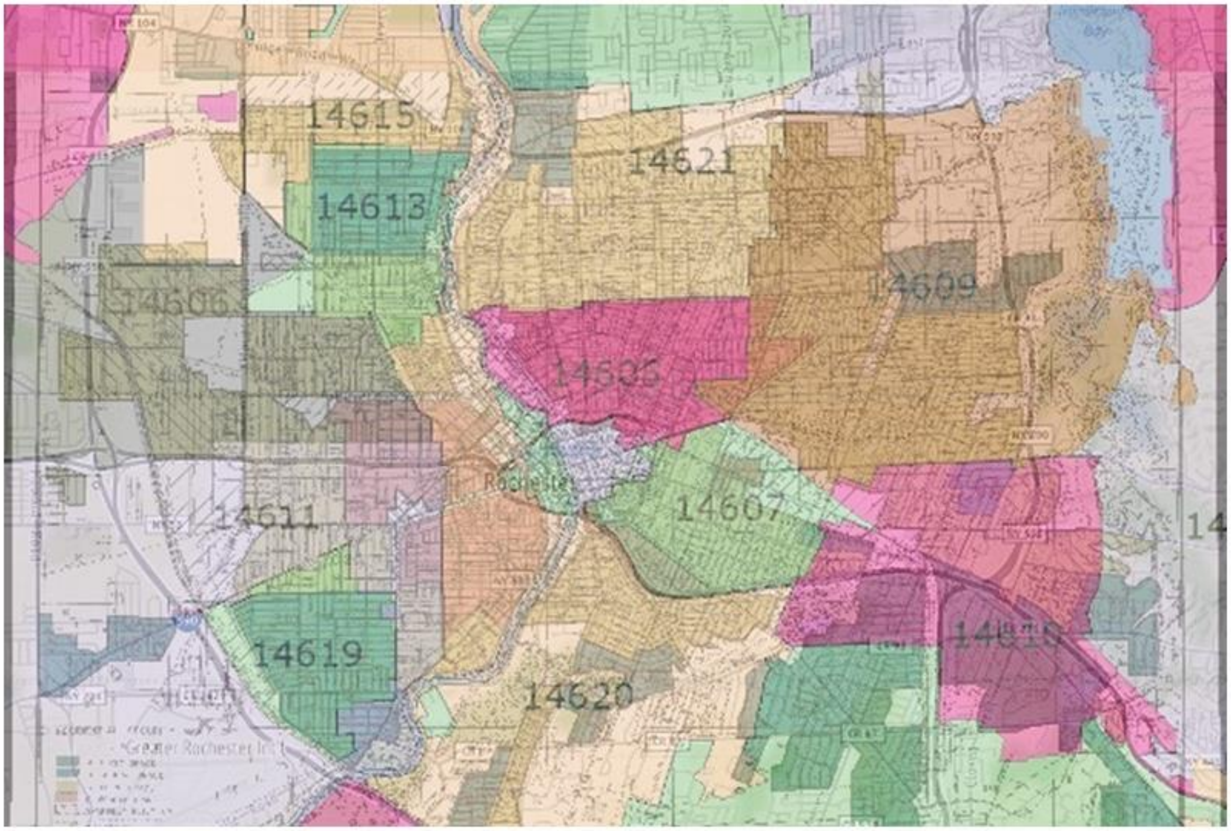

© 2021 Hollenbach SJ et al. *JAMA Network Open.*

eFigure 2. Distribution of Community Resources

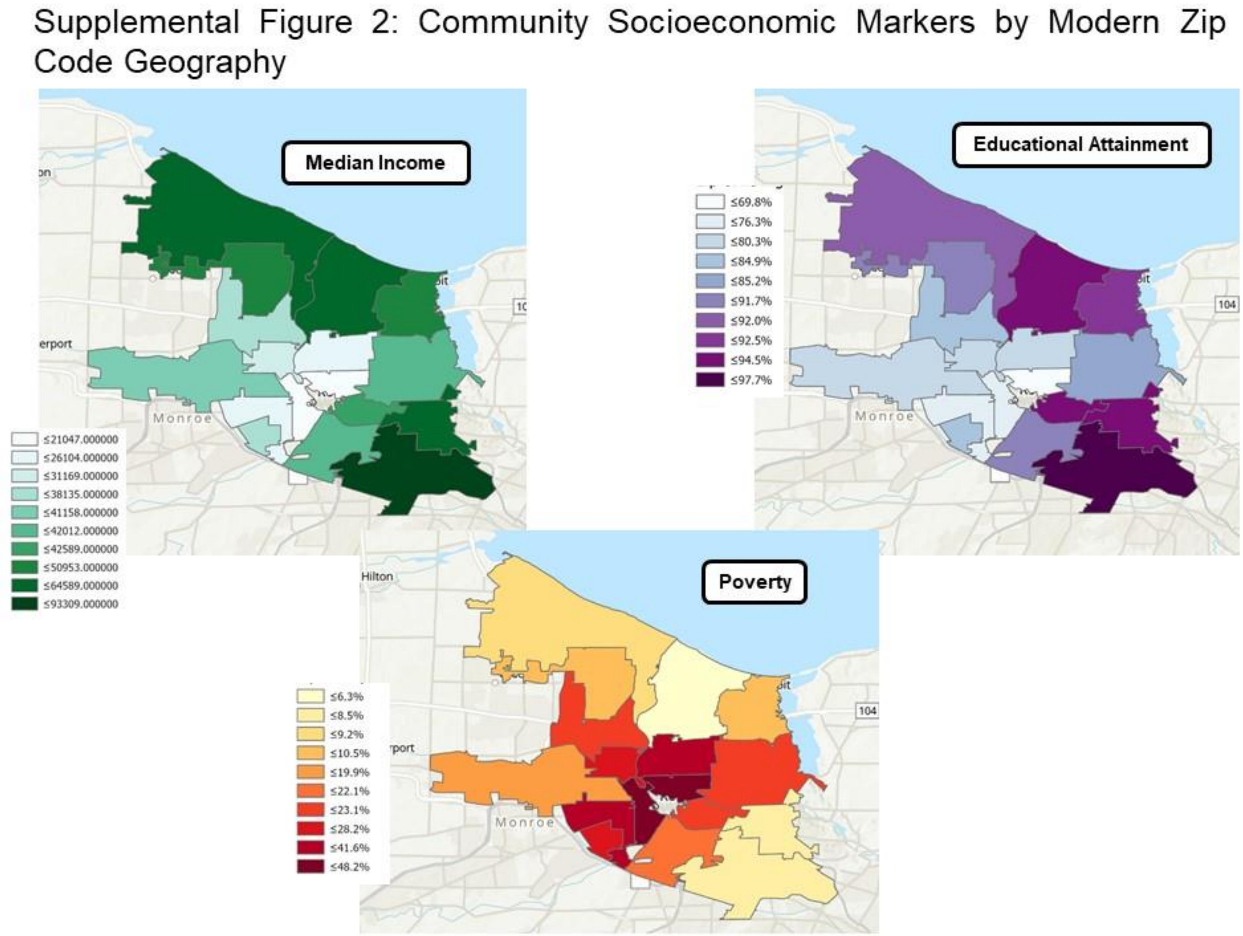

eFigure 2: Visual representation of distribution of community resources as defined by the US Census Bureau data (American Families Survey) 2017 for median income in USD (left), attainment of a high school diploma (right), and percentage of those living below the Federal Poverty Level (bottom).

© 2021 Hollenbach SJ et al. *JAMA Network Open.*

eFigure 3. Incidence of Any Preterm Birth as by Historic HOLC Designation

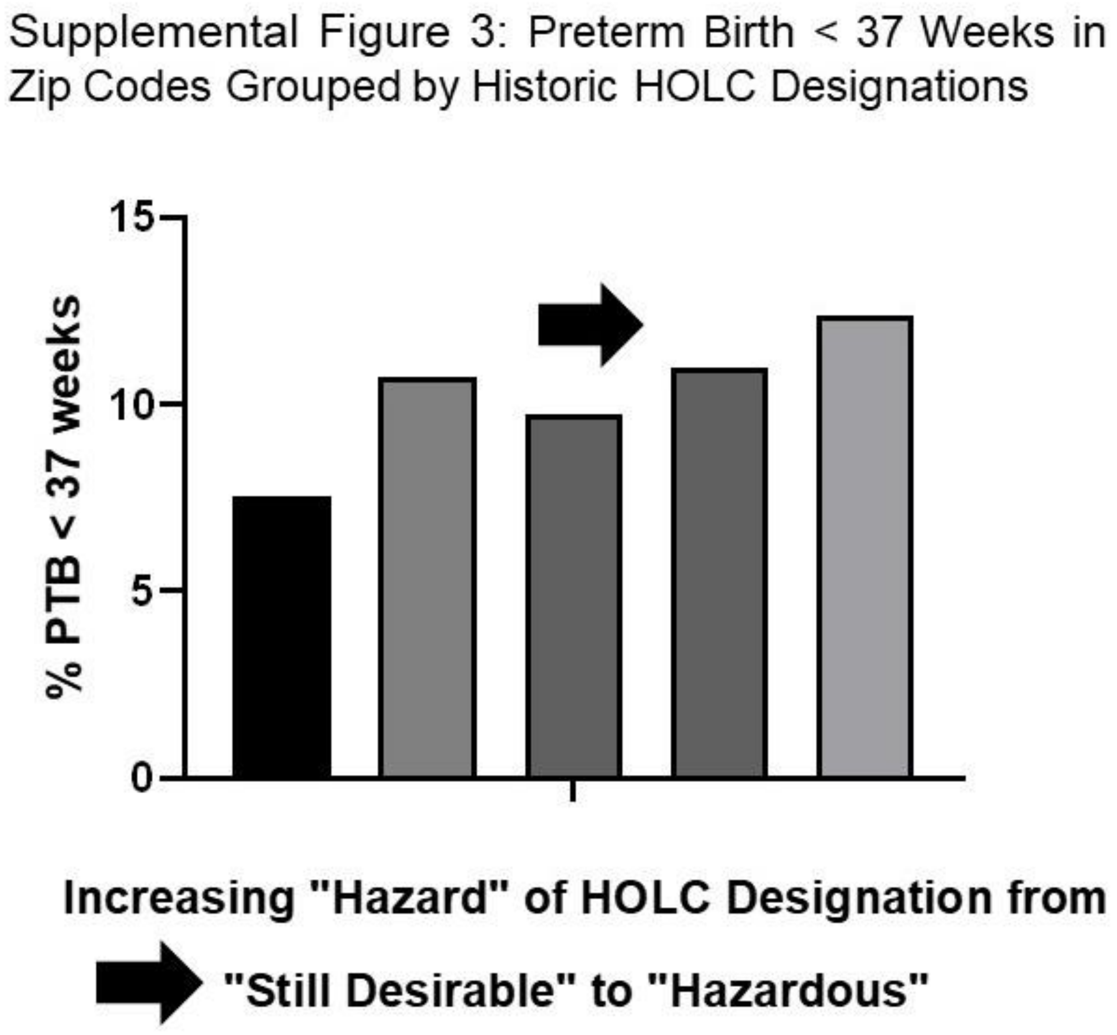

eFigure 3: Graphic representation of the incidence of any preterm birth as the “Hazard” of historic designation increased from “Best” to “Hazardous.”

© 2021 Hollenbach SJ et al. *JAMA Network Open.*

eFigure 4. Incidence of Extreme Preterm Birth and Periviable Birth by Historic HOLC Designation

Supplemental Figure 4: Extremely Preterm Birth in Zip Codes Grouped by Historic HOLC Designations

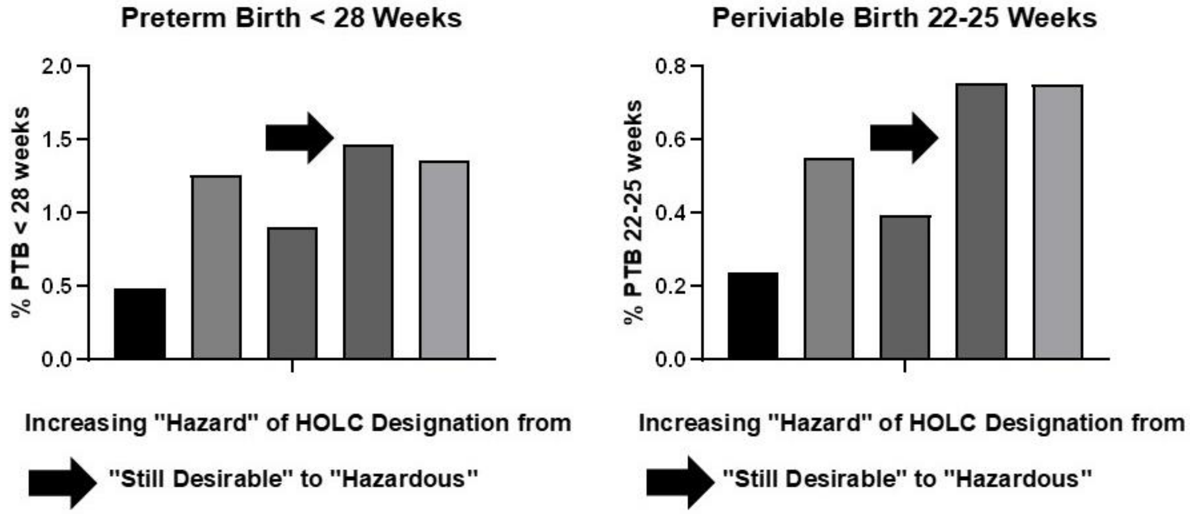

eFigure 4: Graphic representation of the incidence of extreme preterm birth less than 28 weeks and periviable birth less than 26 weeks as the “Hazard” of historic designation increased from “Best” to “Hazardous.”

© 2021 Hollenbach SJ et al. *JAMA Network Open.*
